# Gastrodin ameliorates the lipopolysaccharide-induced neuroinflammation in mice by downregulating miR-107-3p

**DOI:** 10.3389/fphar.2022.1044375

**Published:** 2022-12-08

**Authors:** Jin-Jin Song, Hui Li, Nan Wang, Xiao-Yan Zhou, Yan Liu, Zhen Zhang, Qian Feng, Yu-Ling Chen, Dan Liu, Jia Liang, Xiang-Yu Ma, Xiang-Ru Wen, Yan-Yan Fu

**Affiliations:** ^1^ Department of Genetics, Key Laboratory of Genetic Foundation and Clinical Application, Xuzhou Engineering Research Center of Medical Genetics and Transformation, Xuzhou Medical University, Xuzhou, Jiangsu, China; ^2^ Jiangsu Key Laboratory of Brain Disease and Bioinformation, Research Center for Biochemistry and Molecular Biology, Xuzhou Medical University, Xuzhou, China; ^3^ Clinical Laboratory, Affiliated Hospital of Xuzhou Medical University, Xuzhou, Jiangsu, China; ^4^ College of Pharmacy, Xuzhou Medical University, Xuzhou, Jiangsu, China; ^5^ Department of Cell Biology and Neurobiology, Xuzhou Medical University, Xuzhou, Jiangsu, China

**Keywords:** miR-107-3p, gastrodin, neuroinflammation, depressive-anxiety-like behaviors, lipopolysaccharide

## Abstract

**Background:** Neuroinflammation plays a pivotal role in the pathogenesis of Central Nervous System (CNS) diseases. The phenolic glucoside gastrodin (GAS), has been known to treat CNS disorders by exerting anti-inflammatory activities. Our aim was to investigate the potential neuroprotective mechanisms of GAS on lipopolysaccharide (LPS)-induced mice.

**Methods:** Male C57BL/6J mice were treated by LPS, before which GAS was adminisrated. The behavior tests such as forced swim test, tail suspension test, and elevated plus maze were performed to evaluate depressive-anxiety-like behaviors. A high-throughput sequencing (HTS) analysis was performed to screen out distinctive miRNAs which were validated using quantitative real-time PCR. Then, miRNA agomir or NC was injected stereotaxically into hippocampus of mice to explore the role of miRNA on GAS in response to LPS. Furthermore, Immunofluorescence and the hematoxylin and eosin (H&E) staining were employed to observe the cellular morphology. The protein levels of pro-inflammatory factors were evaluated by western blot. Finally, the target mRNA of miRNA was predicted using bioinformatics analysis. GO and KEGG enrichment analyses were conducted to clarify the potential function of target protein, which were visualized by bubble charts.

**Results:** The behavioral data showed that mice in the LPS group had obvious depressive-anxiety-like behaviors, and 100 mg/kg GAS could improve these behavioral changes and alleviate the levels of pro-inflammatory cytokines in the hippocampus when mice were exposed to LPS for 6 h. Meanwhile, LPS-induced microglia and astrocyte activation in the CA1, CA2, CA3, and DG regions of the hippocampus were also reversed by GAS. Furthermore, miR-107-3p were screened out and verified for GAS in response to LPS. Importantly, miR-107-3p overexpression negatively abrogated the neuroprotective effects of GAS. Moreover, KPNA1 might be the target molecular of miR-107-3p. KPNA1 might regulate 12 neuroinflammation-related genes, which were mainly involved in cytokine−mediated signaling pathway.

**Conclusion:** These results suggested that GAS might alleviate the LPS-induced neuroinflammation and depressive-anxiety-like behaviors in mice by downregulating miR-107-3p and upregulating the downstream target KPNA1. The indicates miR-107-3p may provide a new strategy for the treatment of CNS diseases.

## Introduction

Central Nervous System (CNS) diseases are common clinical disorders characterized by structural or functional abnormalities of the brain or spinal cord (Kundap et al., 2017), such as Parkinson’s, Alzheimer and stroke. Recently, they have been drawn considerable attention worldwide with high morbidity and its possible sequelaes, especially depression and anxiety, which lead to an increase in families and social-economic burden (Liu et al., 2018). Hence, deep understanding the pathogenesis of CNS diseases accompanied by depression becomes a more pressing priority than ever. Neuroinflammation is now recognized as one of the most shared pathologies in multiple CNS disorders (Mithaiwala et al., 2021). In the brain, the inflammatory response is triggered by continuously activation of widely distributed microglia and/or astrocytes during the pathological process (Liang et al., 2019). Overproduction of pro-inflammatory cytokines such as TNF-α, IL-1β, and IL-6 eventually leads to a series of behavioral changes by promoting neuron damage (Kempuraj et al., 2016). What’s more, mounting evidence shows that neuroinflammation can be responsible for multiple psychiatric disorders, such as depression and anxiety (Radtke et al., 2017). Accordingly, anti-neuroinflammatory could be an effective strategy for the treatment of CNS diseases and their complications. Currently, there is still lack of precise and effective medicine for the corresponding diseases clinically. However, traditional Chinese medicine has been widely used in the treatment of the diseases due to its advantages of good efficacy and few side effects, and it provides broad application prospects in the prevention and treatment of CNS diseases related to neuroinflammation.

Gastrodin (GAS), a phenolic glucoside, derived from the rhizoma of Gastrodintrodia elata Blum, has been considered a potentially ideal drug for some inflammatory-related brain diseases owing to its efficacy and safety (Zhang J. S. et al., 2016). As a major bioactive component from the Chinese herbal medicine Gastrodintrodia elata Blum, GAS has been traditionally used to treat various disorders such as headache, dizziness, spasm, epilepsy, stroke, and amnesia due to its anti-oxidant and anti-inflammatory properties (Liu et al., 2018). Notably, in light of its higher absorption rate and permeation through the blood-brain barrier into the brain, GAS has been reported to treat brain diseases such as brain ischemia (Xiao et al., 2021), Alzheimer’s disease (AD) (Shi et al., 2020), or Parkinson’s disease (PD) (He et al., 2021). As discussed above, GAS showed beneficial influences on the brain. However, the underlying mechanism of GAS on the neuroprotective effects has not been clearly elucidated. A series of studies indicate that GAS could modulate NF-kB, MAPKs, and Notch-1 signaling pathways in brain inflammatory processes (Dai et al., 2011; Yao et al., 2022). Others believe that GAS could regulate STAT3 signal pathways to inhibit inflammasome in reactive astrocytes (Sui et al., 2019). In addition, it is reported that GAS could ameliorate depressive-like behaviors by upregulating the expression of BDNF in the hippocampus (Zhang et al., 2014). To date, the precise molecular mechanism of GAS on antineuroinflammation and antidepressant and antianxiety effects remains unclear.

MicroRNAs (miRNAs/miRs), the small endogenous non-coding RNAs consisting of 20–25 nucleotides, regulate a variety of biological processes through pairing-based at the 3′ end of the noncoding region of the target messenger RNAs (mRNAs) (Hammond, 2015). It has been widely reported that miRNAs can regulate the development and function of the brain (Sempere et al., 2004). Dysregulation of miRNAs has been shown to contribute to a variety of CNS disease processes (Brites and Fernandes, 2015; Peng et al., 2020). Furthermore, miRNAs are regarded as promising biomarkers due to their stable and easy reproducible detection (Wang et al., 2018; Xu et al., 2019). Studies have also shown that miRNAs such as miR-144 and miR-107 are involved in biological mechanisms of Chinese herbal therapy for brain diseases (Chu et al., 2019; Cheng et al., 2020). Thus far, miRNAs plays an important role in CNS related diseases and have been an area of intense investigation. Recently, high-throughput sequencing (HTS) has been developed for sequence-based miRNAs identification (Zhao et al., 2010). Compared with microarray, HTS has been shown to have pronounced merits for the discovery of small RNA. So far, HTS has gained widespread adoption for global miRNA expression profiling in the brain (Kong et al., 2015; Wang et al., 2020). Therefore, HTS has gradually been widely used to identify biomarkers and therapeutic targets under different conditions (Lai et al., 2011; Carlson et al., 2013). However, the role of miRNA remains largely unexamined in GAS intervention on LPS-induced neuroinflammation in the mice, as determined by HTS method.

Herein, the purpose of this study was to examine the effects of GAS on LPS-induced neuroinflammation and depressive-anxiety-like behaviors in mice, then screen out distinctive miRNAs. Moreover, we used miRNA agomir to explore the therapeutic effects of GAS on neuroinflammation related to CNS diseases *via* miRNA pathway.

## Materials and methods

### Animals

Adult male C57BL/6 mice (24 ± 2 g) were purchased from Pengyue Laboratory Animal Breeding Co., Ltd., (Jinan, China). Mice were allowed *ad libitum* access to food and water and were maintained at 25°C ± 2°C with a 12-h light/12-h dark cycle. All procedures were performed in accordance with the guidelines for the use and care of laboratory animals and approved by the committee of experimental animal administration of the Xuzhou Medical University. The animals were acclimatized for 3 days before the experiment.

### Reagents and antibodies

LPS (from *Escherichia coli* 055:B5) was purchased from Sigma (St. Louis, MO, United States). Gastrodin (purity: 99%) was obtained from Shanghai Aladdin Biochemical Technology Co., Ltd. The following primary antibodies were obtained from Proteintech: TNF-α, IL-1β, IL-18, IL-6, and MCP-1. NeuN, IBA1, and GFAP antibodies were obtained from Abcam, Wako, and Cell Signaling, respectively. miRNA-107-3P agomir was synthesized by Sangon Biotech (Shanghai, China). The secondary antibodies were purchased from Sigma.

### Drug administration

LPS and gastrodin were all dissolved in sterile saline. After adaptation, the mice were randomly divided into five groups: saline group, LPS group, and three LPS + GAS (50, 100, or 200 mg/kg) groups. All of the mice, except for those in the saline group, were intraperitoneally injected with a single dose of 1 mg/kg LPS. Concurrently, 50, 100, and 200 mg/kg GAS were given by intraperitoneal injection as low, middle, and high dose GAS groups, respectively. Subsequently, mice were treated with different concentrations of GAS (50 mg/kg, 100 mg/kg, and 200 mg/kg) 1 h after LPS stimulation. The saline group only received an equal volume of 0.9% saline. After HTS of miRNAs, the mice were divided into two groups: an LPS + GAS + miRNA-107-3p agomir group and an LPS + GAS + agomir NC group. The two groups were stereotactically injected into hippocampal CA1 region of mice.

### Experimental schedule

The animal behaviors tests, namely Open-Field Test (OFT), Closed-Field Test (CFT), Forced swim test (FST), Tail suspension test (TST), and Elevated plus maze test were measured at 6 and 24 h after LPS and GAS administration. Afterward, the mice of saline, LPS, and 100 mg/kg GAS groups were sacrificed and the brain tissues were harvested for miRNA, protein, and morphological analyses. The mice of the LPS + GAS + miRNA-107-3p group and the LPS + GAS + NC group were also subjected to behavioral tests, namely TST, FST, OFT, CFT, and EPM, and subsequently sacrificed to be used for mRNA and protein analyses. The entire experimental schedule is shown in Figure 2.

### Behaviors tests

#### Locomotive activity

OFT and CFT were carried out to evaluate the spontaneous locomotive activity of the mice according to the previously report^25^. Each mouse was placed in the center of an open field or a closed-field apparatus (W50 cm^3^ × D50 cm^3^ × H30 cm^3^) to acclimate for 3 min, followed by recording their free moving behaviors for 5 min using the ANY-maze Video Tracking System. The total distance traveled and the crossing lines were analyzed.

#### Forced swim test

Learned helplessness or behavioral despair was evaluated with the FST as previously described (Zhang et al., 2016). Mice were given 6 min to swim. Afterward, the swimming time and immobility time were recorded. Immobility was defined as the absence of any movement except for the head above water.

#### Tail suspension test

Learned helplessness or behavioral despair was also assessed with the TST based on our previously described (Zhang et al., 2016). Each mouse was hung upside down by its tail, with its head 5 cm from the bottom. The time was recorded for a 6 min session. Immobility was defined as the absence of any limb or body movements besides respiration.

## Elevated plus maze test

The EPM test was applied according to a previously described method (Beheshti et al., 2020). Briefly, mice were placed on the center platform of the EPM, which consists of four arms (49 cm in length, 10 cm in width, and 50 cm in height each arm) in the shape of a plus sign. The movements of the animal were observed and scored for 5 min. The ratio of the total times remained in the open and closed arms was used to calculate.

### Immunofluorescence staining

To detect of intact neuron, microglia, and astrocyte in the hippocampus, immunofluorescence staining was adopted. Briefly, after the behavioral tests, upon anesthesia, the mice were perfused transcardially. Following this, mice brains were immediately fixed with 4% paraformaldehyde solution and sunk into sucrose solution. After this, midbrain tissue was frozen and embedded with OCT embedding medium followed by cutting into 30-μm-thick coronal sections. The sections were washed thrice with 1% PBS after thawing at room temperature for 30 min. The brain slices were blocked with blocking buffer (1% BSA in TBST) for 30 min at 4°C followed by incubation with the combination of primary antibodies such as anti-GFAP (1:400), anti-Iba1 (1:1000), anti-NeuN (1:500) for overnight at 4°C. After washing, appropriate secondary antibodies conjugated with fluorescent were then incubated for 2 h at 37°C. Afterward, the slices were stained with DAPI at room temperature for 10 min after washing. The mean fluorescence intensity (MFI) was quantified using ImageJ software.

### Hematoxylin-eosin staining

HE staining was conducted to evaluate the protective effects of GAS on neuronal loss and damage in the hippocampus of LPS-induced mice. Excised midbrain specimens were immersed in 4% paraformaldehyde for 24 h and dehydrated in an alcohol series embedded in paraffin, then cut into tissue sections (3 µm thick). Then the sections were deparaffinized by xylene, and xylene was removed by alcohol. Finally, tissue sections were stained with H&E. Light microscopy was used to observe the morphology.

### Western blot assay

WB was performed to further determine the effects of GAS on neuroinflammation as in our previous report (Fu et al., 2015). Briefly, the proteins were obtained from the hippocampal tissues and the protein content was quantified using BCA. Equal amounts of protein were separated with 8%–12% SDS-PAGE gels and then electro-transferred onto a nitrocellulose membrane (NC). The membranes were blocked with 5% skim milk in Tris-buffered saline (TBS) for 1 h and then incubated overnight at 4°C with specific primary antibodies (anti-TNF-α, anti-IL-1β, anti-IL-18, anti-IL-6, and anti-MCP-1) (1:1000). After washing, membranes were incubated with fluorescence secondary antibodies for 2 h at room temperature. The bands on the membrane were scanned and analyzed using ImageJ software.

### High-throughput sequencing analysis

To further elucidate the role of miRNAs, HTS was used to screen differential miRNAs. Mice hippocampus from the saline group, the LPS group, and the LPS + GAS group were removed to analyze using HTS which was completed by Huada Biotechnology Co. Significant differentially expressed miRNAs were collected based on the Q ≤ 0.05, |log2 fold change| ≥ 1 as the threshold value filtering. Subsequently, volcano plots were drawn, in which red represented high expression and green represented low expression. Further, the changes of differentially expressed miRNAs were drawn into a systematic clustering heat map, with red representing high expression and green representing low expression. The raw data from HTS were uploaded to the GEO database (GSE214909).

### Stereotaxic injection of miR-107-3p

To ascertain the role of miR-107-3p on GAS intervention after LPS treatment, overexpression miR-107-3p was utilized by miR-107-3p agomir. Mice were anesthetized and placed in an animal stereotaxic frame. The injection coordinates relative to bregma were as follows: 1.82 mm posterior, 1.30 mm lateral, and 1.86 mm ventral. A total of 0.2 μl miR-107-3p agomir or agomir NC was injected into the hipocampal CA1 region. Subsequent experiments were performed 12 h after injection.

### Prediction of target moleculars

The putative targets of miRNAs were predicted using three different commonly used databases: TargetScan (http://www.targetscan.org/vert_80/), miRWalk (http://129.206.7.150/search_mirnas/) and miRTar2GO (http://www.mirtar2go.org/). After selecting the results based on their indexes, the overlapping interactions were presented as a Venn diagram constructed using a web-based tool (https://bioinfogp.cnb.csic.es/tools/venny/index.html). Then GTExPortal software GTEx (https://gtexportal.org/home/) was used to query the distribution in tissues and organs.

### qRT-PCR for differential miRNA and predicted target moleculars

qRT-PCR was used to validate several miRNAs differentially expressed among the saline group, the LPS group, and the LPS + GAS group. In brief, RNAs were isolated and cDNA was reversely transcribed using miRcute miRNA cDNA Synthesis Kit (Tiangen Biotechnology Co). The reaction procedure was performed as follows: 3 min at 95°C, and then 45 cycles of denaturation at 95°C for 5 s and annealing/extension at 60°C for 30 s. Meanwhile, to determine whether there were changes in the expression of targets, qRT-PCR was also used to quantify the target mRNA expression level of miR-107-3p.

The qPCR reaction was carried out with the SYBRGreenER™ Kit (Invitrogen) according to the manufacturer’s instructions with three replicates. The process was performed on the LightCycle 480II (Roche, Rotkreuz, Switzerland). The relative expression was calculated and normalized using the 2^−ΔΔCT^ method. U6 and β-actin were utilized as an endogenous control for miRNAs and mRNA, respectively. The primers used in the current study are listed in Supplementary Table S1.

### Protein-protein interaction network construction and intersection targets of KPNA1 and neuroinflammation

The NCBI common database (https://www.ncbi.nlm.nih.gov/) was performed to extract the mouse derived proteins by retrieving “neuroinflammation” term. The STRING database (https://string-db.org/) was used to compile the protein–protein interaction (PPI) network of potential target genes of KPNA1, with the species limited to mouse (Mus musculus) and the interaction score ≥0.9 with highest confidence. Cytoscape software (version 3.8.2) were peformed to visualize the PPI network. Then, the intersection was obtained by overlapping the predicted targets of KPNA1 and the target genes related to neuroinflammation through Venny2.1 software.

### GO enrichment analysis and kyoto encyclopedia of genes and genomes pathway enrichment analysis

To investigate the common target genes of KPNA1 and neuroinflammation, GO function and KEGG pathway were analyzed with Enrichr (https://maayanlab.cloud/Enrichr/). GO enrichment was analyzed from the perspective of biological process, cellular component, and molecular function. Bubble plots were drawn using the GGplot2 package of R language according to the enrichment degree.

### Correlation analysis

To explore KPNA1 on the neuroinflammation, the correlation of KPNA1 with common inflammatory factors TNF, IL-6, IL-1β, IL-18, MCP1 in the hippocampus, respectively, was analyzed with Pearson correlation coefficients using the correlation analysis tool of GEPIA based on data from GTEx. GTEx Portal database “Brain-Hippocampus” and Correlation Coefficient Pearson were used to analyse the correlations.

### Statistical analysis

All of the values are expressed as the means ± SEM. Statistical analyses were performed using the SPSS 20.0. One-way analysis of variance (ANOVA) followed by least significant difference (LSD) post hoc multiple comparisons among multiple groups. A value of *p* < 0.05 was regarded as statistically significant.

## Results

### Gastrodin ameliorates lipopolysaccharide-induced depressive-anxiety-like behaviors in mice

**FIGURE 1:**
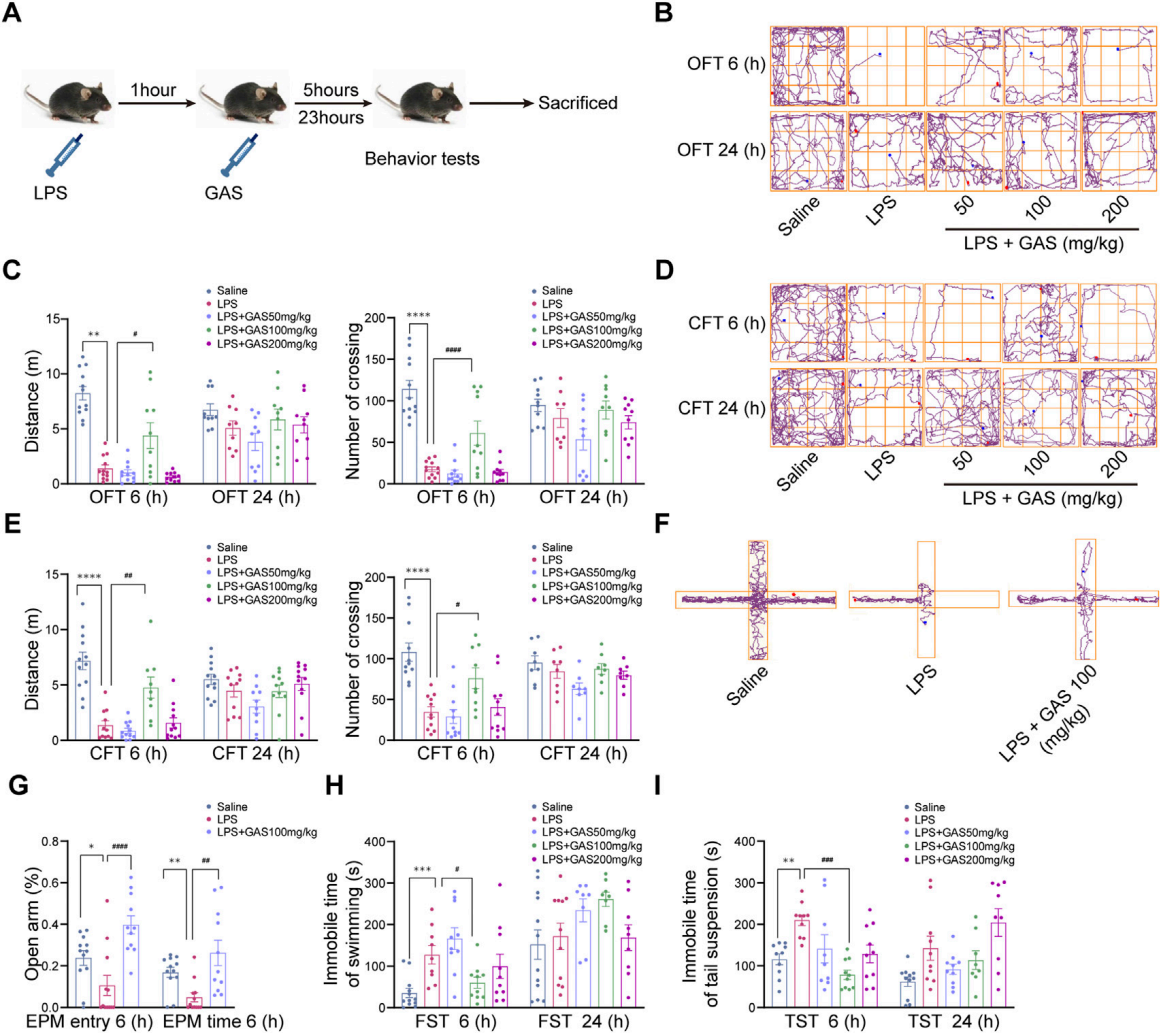
Effects of GAS on locomotor activity and depressive-anxiety- like behaviors after 6 and 24 h of LPS treated mice. **(A)** This part of the experimental flow chart. **(B,C)** The track maps, total distance traveled, and crossing lines during 5 min in the OFT. **(D,E)** The track maps, total distance traveled, and crossing lines during 5 min in the CFT. **(F,G)** The track maps, the ratio of the entry and time spent in the open arms in the EPM. **(H)** The immobile time of swimming for 6 min in the FST. **(I)** The immobile time of suspension during 6 min in the TST. All data are expressed as means ± SEM for twelve mice in each group. ****p < 0.0001, ***p < 0.001, **p < 0.01, *p < 0.05 versus respective saline group; ^####^ *p* < 0.0001, ^###^ *p* < 0.001, ^##^ *p* < 0.01, ^#^ *p* < 0.05 versus LPS group. LPS, lipopolysaccharide; GAS, gastrodin; OFT, open-field test; CFT, close-field test; FST, forced swimming test; TST, tail suspension test; EPM, elevated plus maze.

Mice were injected intraperitoneally with 1 mg/kg LPS to induce neuroinflammation and depressive-like behavior followed with GAS medication. Then, the OFT, CFT, FST, and TST were performed to observe the effects of GAS on depressive-like behaviors at 6 and 24 h. This part of the experimental flow chart was shown in Figure 1A. As shown in Figures 1B–E, after LPS induction modeling, the total distance traveled and the number of the crossing of mice 6 h had a significant drop in the OFT and CFT compared with the saline group, while the middle-dose GAS treatment group were significantly increased compared with the LPS group. Meanwhile, the EPM results for testing anxiety-like behavior (Figures 1F,G) indicating that GAS could reverse the LPS-induced reduction in the percentage of the time and entry spent in the open arms. Secondly, for the TST and FST, it was shown that the mice in the LPS group exhibited significantly longer immobility time at 6 h after LPS injection, and the middle-dose GAS ameliorated this depressive-like behavior in comparison with the LPS group (Figures 1H,I). However, Upon exposure to LPS for 24 h, all groups had no significant differences in all the above behavioral tests. Above all, these observations demonstrated that 100 mg/kg GAS significantly relieved depressive-anxiety-like behaviors at 6 h after LPS injection to present antidepressant and antianxiety properties. Based on these results, 100 mg/kg GAS was selected for further mechanistic studies.

### Gastrodin attenuates lipopolysaccharide-induced neuroinflammation in mice

**FIGURE 2:**
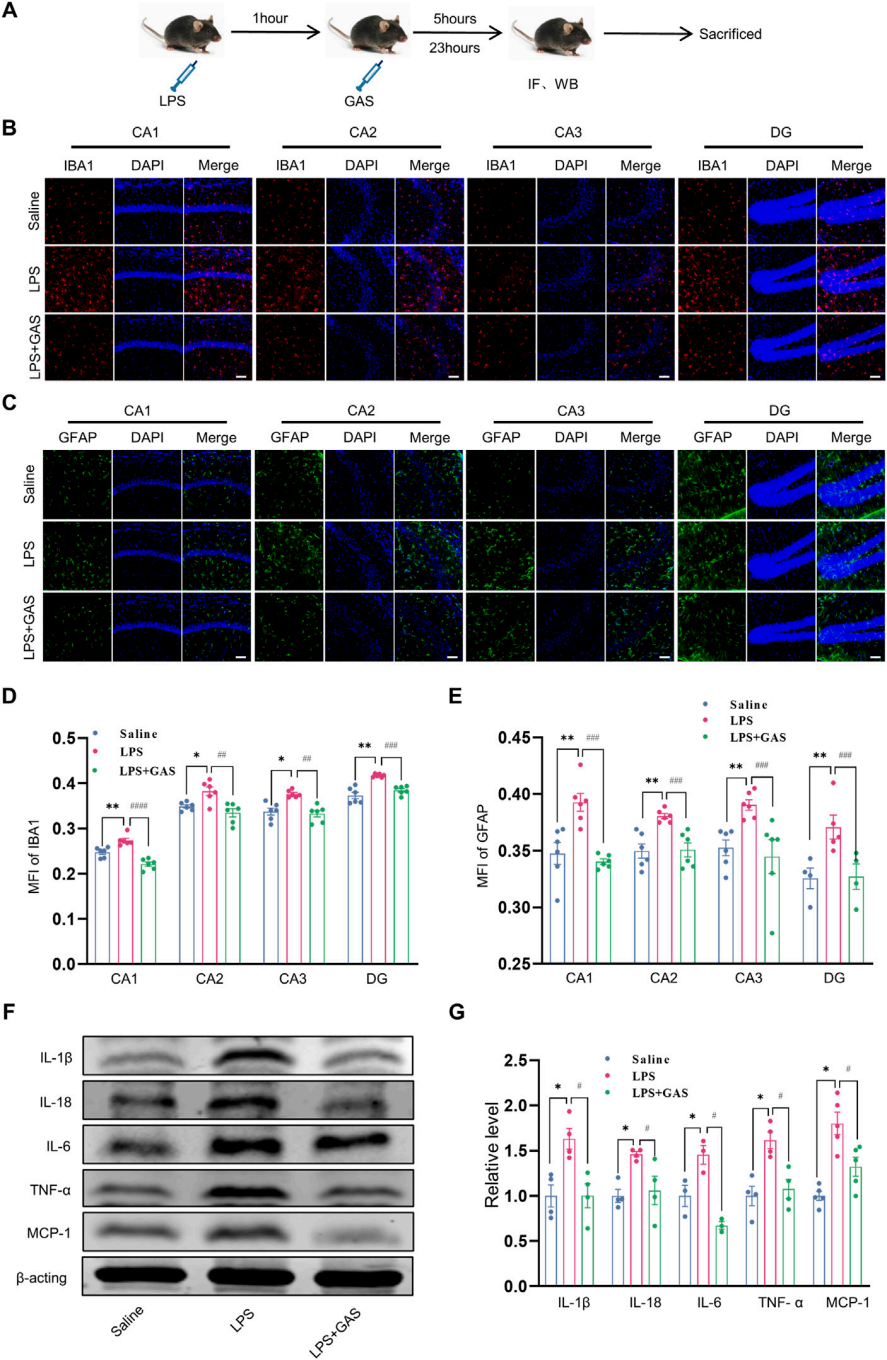
Effects of GAS on neuroinflammation in the hippocampi of mice. **(A)** This part of the experimental flow chart. **(B–E)** representative images of IF staining and histograms of MFI for GFAP-positive astrocyte (green), and IBA1-positive microglia (red) in the CA1, CA2, CA3, and DG regions of hippocampus. Scale bar = 30 μm. **(F,G)** Representative protein bands and the histograms of bands intensity analysis of IL-1β, IL-18, IL-6, TNF-α, and MCP-1 in the hippocampus. Values are represented as means ± SEM for three mice in each group. ***p < 0.001, **p < 0.01, *p < 0.05 versus saline group; ^###^ *p* < 0.001, ^##^ *p* < 0.01, ^#^ *p* < 0.05 versus LPS group. LPS, lipopolysaccharide; GAS, gastrodin; IL-1β, Interleukin-1; IL-6, Interleukin-6; IL-18, Interleukin-18; TNF-α, Tumor necrosis factor-α; MCP-1, Monocyte Chemoattractant Protein-1; IF, Immunofluorescence; WB, western blot; MFI, the mean fluorescence intensity.

To ascertain the roles of GAS on neuroinflammation, the IF, HE staining and WB methods were used to measure inflammatory response in hippocampus. This part of the experimental flow chart was shown in Figure 2A. The MFI of IBA1-labeled microglia in the CA1, CA2, CA3, and DG regions of hippocampus, was noticeably increased in the LPS group when compared to the saline group, while the treatment of GAS reversed the LPS-induced the changes in hippocampus (Figures 2B,D). The similar results of GFAP-labeled astrocytes were observed in Figures 2C,E. By contrast, there was no statistical difference in neurons labeled by NeuN (Supplementary Figure S1A). Subsequently, HE staining results also showed no obviously observed morphological change of pyramidal neurons presented in the hippocampus (Supplementary Figure S1B). The results revealed that GAS displayed a significant effect on the activation of microglia and astrocytes in LPS-induced mice. The actions of the cytokines contribute to the response of the brain toward LPS. As the results presented in Figures 2F,G, GAS could mitigate the LPS-induced elevation of IL-1β, IL-18, IL-6, TNF-α, and MCP-1. In summary, these results suggested that GAS might play the therapeutic role by improving LPS-induced inflammation and regulating the function of hippocampal microglia and astrocytes in the hippocampus.

### Gastrodin attenuates may play a neuroinflammation role through miR-107-3p

**FIGURE 3:**
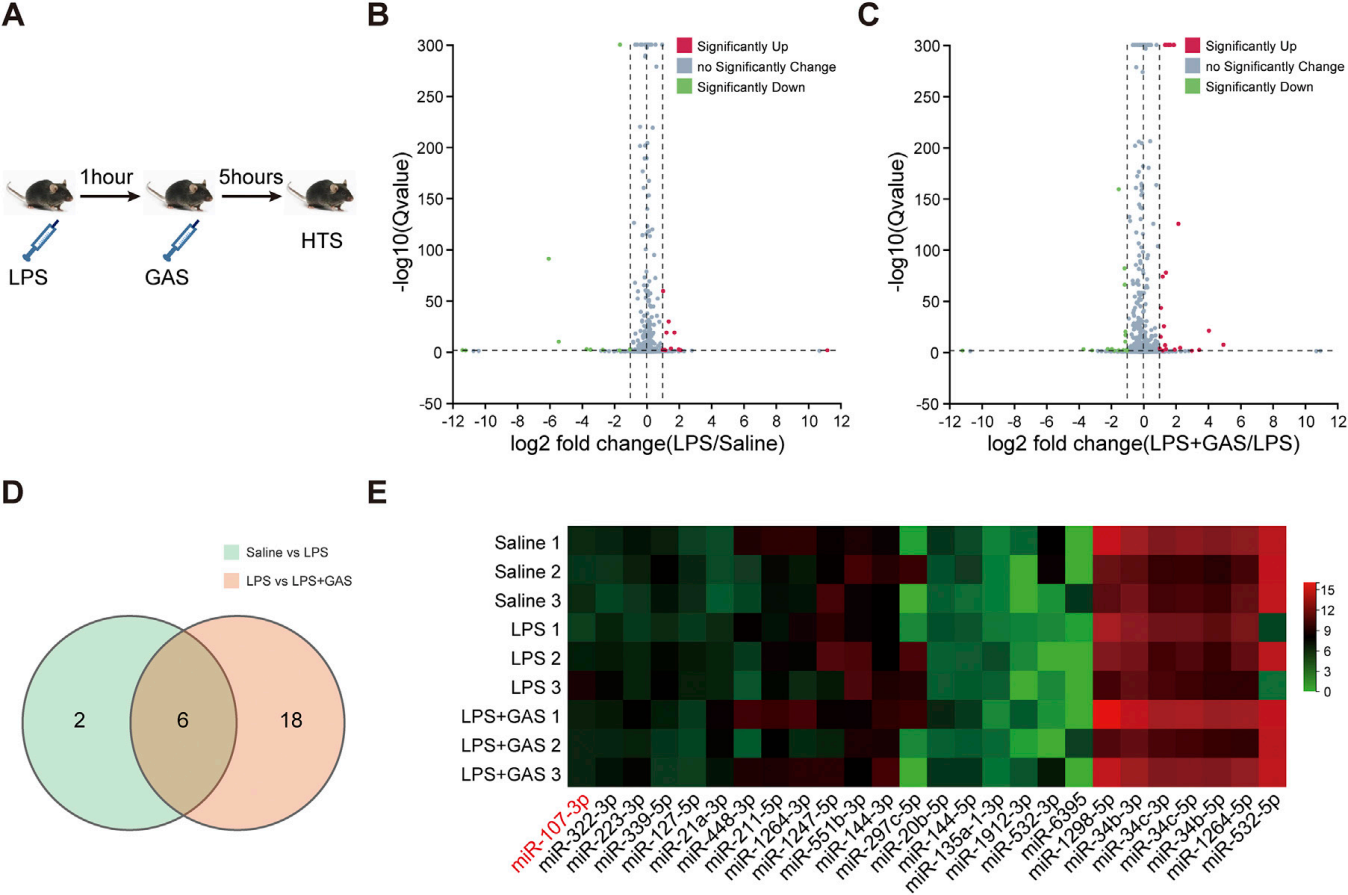
Differential miRNAs profile associated with GAS on LPS induced-neuroinflammation. **(A)** Experimental flow chart of high-throughput sequencing. **(B,C)** Volcano plot based on differently expressed miRNAs (*n* = 3). **(D)** Venn diagram of cluster analysis of significantly differentially expressed miRNAs. **(E)** Cluster analysis heat map of differentially expressed miRNAs. LPS, lipopolysaccharide; GAS, gastrodin.

To investigate distinctive miRNAs which might be associated with the antineuroinflammation and antidepressant and antianxiety mechanism of GAS effects, we used an HTS approach to screen out the changes of the miRNA in the hippocampus (Figure 3A). As showed in Figures 3B–E, we obtained 6 miRNAs (mmu-miR-107-3p, mmu-miR-297-5p, mmu-miR-21a-3p, mmu-miR-532-5p, mmu-miR-532-3p, mmu-miR-6395) which were significantly changed by taking the intersection of 8 putative miRNAs of the LPS group vs. the Saline group and 24 related miRNAs of the LPS + GAS group vs. the LPS group. LPS stimulation induced the expression of miR-107-3p, miR -297-5p, and miR-21a-3p, whereas GAS treatment reduced the expression. However, the expression of miR-532-5p, miR-532-3p, and miR-6395 was opposite after LPS and GAS treatment. These miRNAs were validated the sequencing data by using qRT-PCR. The RNA levels changes of only two miRNAs (miR-107-3p and miR-297-5p) were in line with HTS screening results. In fact, there were no statistical differences. In comparison, the variation of miR-107-3p was more evident in the hippocampus of mice. Finally, miR-107-3p was selected for further study. Therefore, based on the network analysis, we could speculate that miR-107-3p might mediate the effects of GAS on LPS-induced neuroinflammation.

### MiR-107-3p could reverse the effect of gastrodin on lipopolysaccharide-induced depressive-anxiety-like behaviors in mice

Firstly, to detect the diffusion of miR-107-3p in the hippocampus of mice. Bromophenol blue was stereotaxic injected into the hippocampus and lateral ventricles at a fixed point to determine the coordinates of injection (Supplementary Figure S2A). After that, miR-107-3p with green fluorescence label was injected to observe the diffusion in the brain of the mice (Supplementary Figures S2B,C). The results showed that miR-107-3p was well diffused at 12 h after injection of the hippocampus.

**FIGURE 4:**
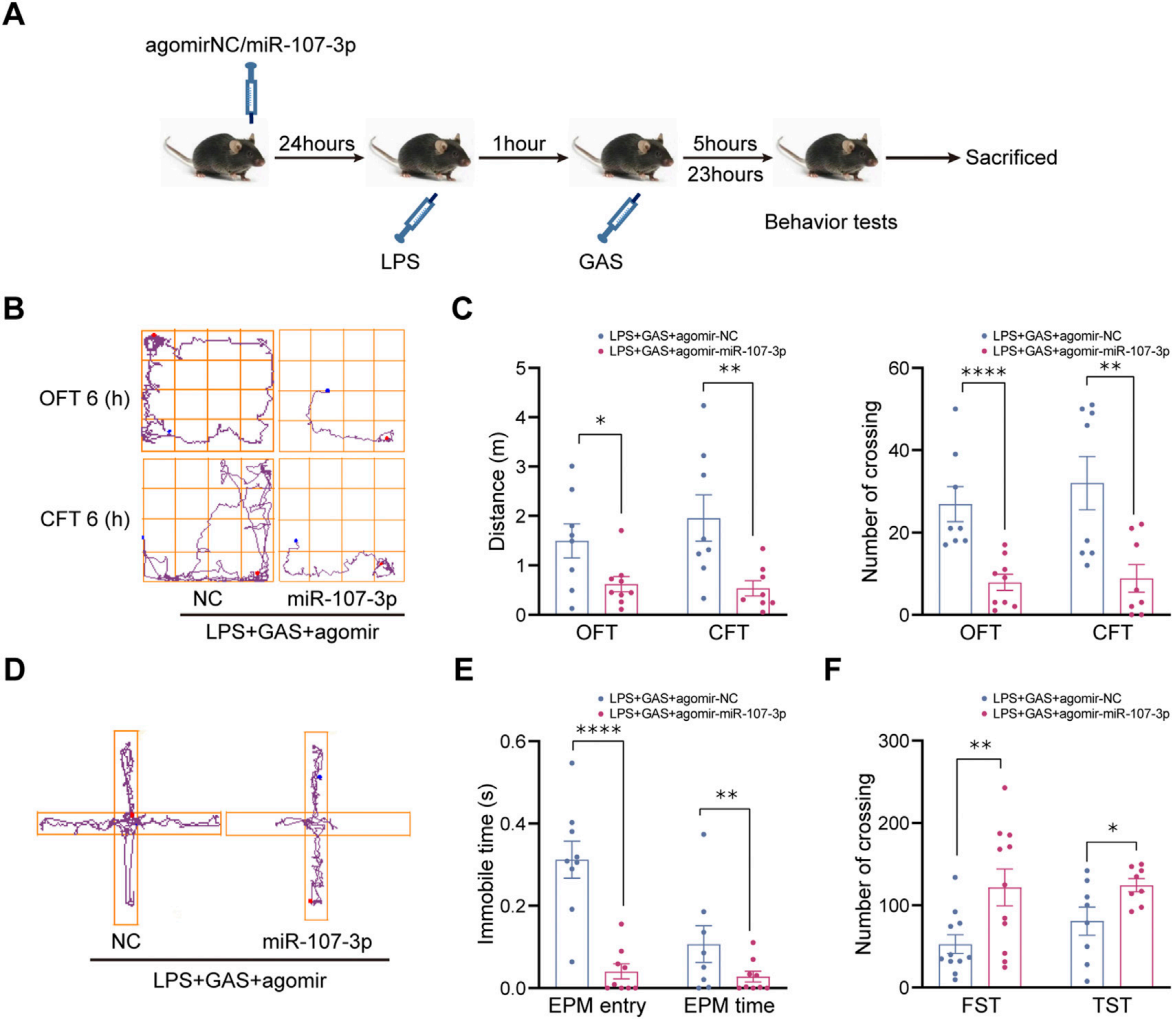
Effects of miR-107-3p on depressive-anxiety- like behaviors after LPS and GAS intervention in mice. **(A)** Flow chart of miR-107-3p agomir injection on the behavioral tests in mice. **(B,C)** The track maps, total distance traveled, and crossing lines in the OFT and CFT. **(D,E)** The track maps, the ratio of the entry and time spent in the open arms in the EPM. **(F)** The immobile time of swimming and suspension in the FST and TST. Values are represented as means ± SEM. *n* ≥ 8 each group. ****p < 0.001, **p < 0.01, *p < 0.05 versus NC group; ^##^ *p* < 0.01, ^#^ *p* < 0.05 versus LPS + GAS + agomir NC group. LPS, lipopolysaccharide; GAS, gastrodin; OFT, open-field test; CFT, close-field test; FST, forced swimming test; TST, tail suspension test; EPM, elevated plus maze.

To further clarify whether miR-107-3p plays a role in gastrodin improving depressive-like behaviors, miR-107-3p agomir (mimics) or agomir NC (control) were injected into the hippocampus of mice (Figure 4A). OFT, CFT, FST, TST, and EPM were used to evaluate the locomotive activity and depressive-anxiety like behaviors 12 h after injection. Compared with the control group, overexpressing miR-107-3p significantly decreased the total distance and number of crossing the lines in OFT and CFT (Figures 4B,C). Also, the similar effects of miR-107-3p were observed in the EPM test, as shown in the comparison between miR-107-3p agomir group and agomir NC group (Figures 4D,E). Likewise, overexpressing miR-107-3p remarkably extended the immobility time in both TST and FST (Figure 4F). These results suggested that miR-107-3p could reverse the antidepressant and antianxiety effect of GAS.

### MiR-107-3p could reverse the anti-inflammatory effects of gastrodin attenuates

**FIGURE 5:**
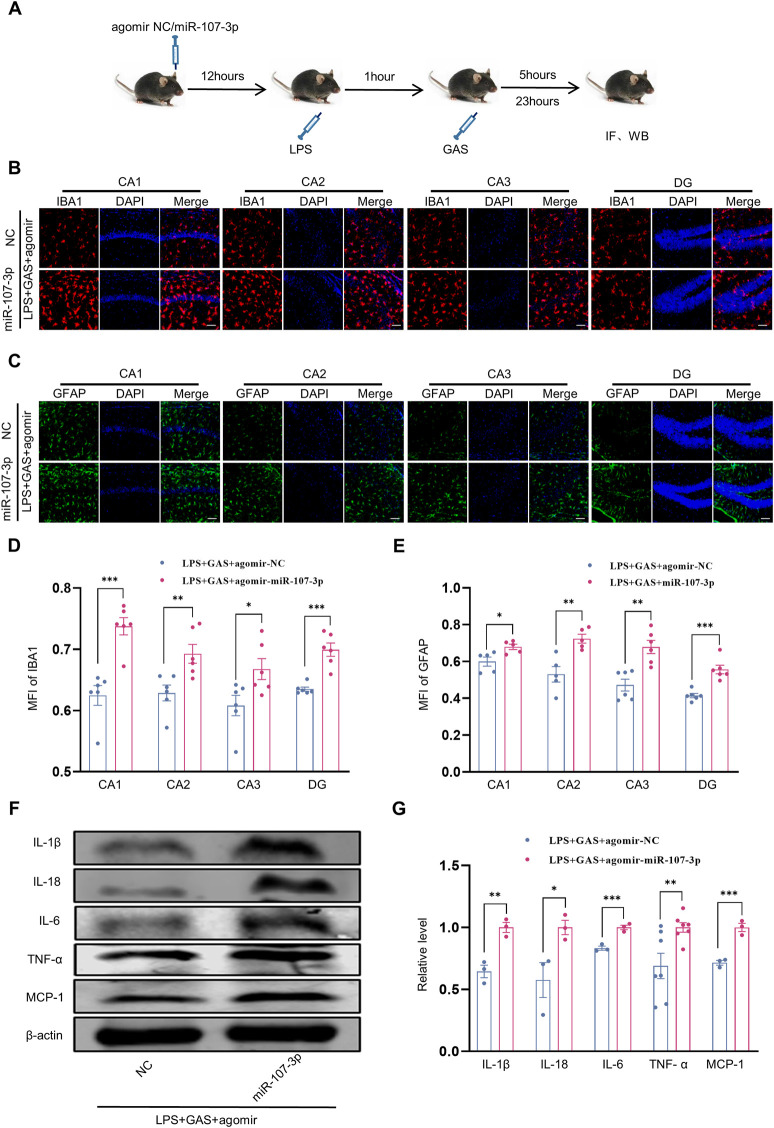
Effects of miR-107-3p on GAS inhibiting the LPS-induced neuroinflammation. **(A)** Flow chart of miR-107-3p agomir injection on the neuroinflammation in mice. **(B–E)** representative images of IF staining and histograms of MFI for GFAP-positive astrocyte (green), and IBA1-positive microglia (red) in the CA1, CA2, CA3, and DG regions of hippocampus. Scale bar = 30 μm. **(F,G)** Representative protein bands and the histograms of bands intensity analysis of IL-1β, IL-18, IL-6, TNF-α, and MCP-1 in the hippocampus. Values are represented as means ± SEM for three mice in each group. ***p < 0.001, **p < 0.01, *p < 0.05 versus LPS + GAS + agomir NC group. LPS, lipopolysaccharide; GAS, gastrodin; IL-1β, Interleukin-1; IL-6, Interleukin-6; IL-18, Interleukin-18; Tumor necrosis factor-α, TNF-α; MCP-1, Monocyte Chemoattractant Protein-1; IF, Immunofluorescence; WB, western blot; MFI, the mean fluorescence intensity.

Furthermore, to explore whether miR-107-3p is involved in GAS inhibiting inflammatory responses, we used WB and IF to assess the expressions of pro-inflammatory factors and the activation of microglia and astrocytes in the hippocampus of mice. The part of the experimental flow chart is shown in Figure 5A. We found that compared with the control group, miR-107-3p overexpression significantly enhanced the MFI of IBA1-labeled microglia and GFAP-labeled astrocytes in all the hippocampal regions, including CA1, CA2, CA3, and DG (Figures 5B–E). However, there was no statistical difference in neurons labeled by NeuN (Supplementary Figure S1C). We also observed that protein expression levels of these pro-inflammatory factors including IL-1β, IL-18, IL-6, TNF-α, and MCP-1 were markedly increased in the LPS + GAS + miR-107-3p agomir group compared with the LPS + GAS + agomir NC group (Figures 5F,G). Taken together, these results demonstrated that miR-107-3p could reverse the anti-inflammatory effects of GAS.

### KPNA1 might be the downstream target of miR-107-3p

**FIGURE 6:**
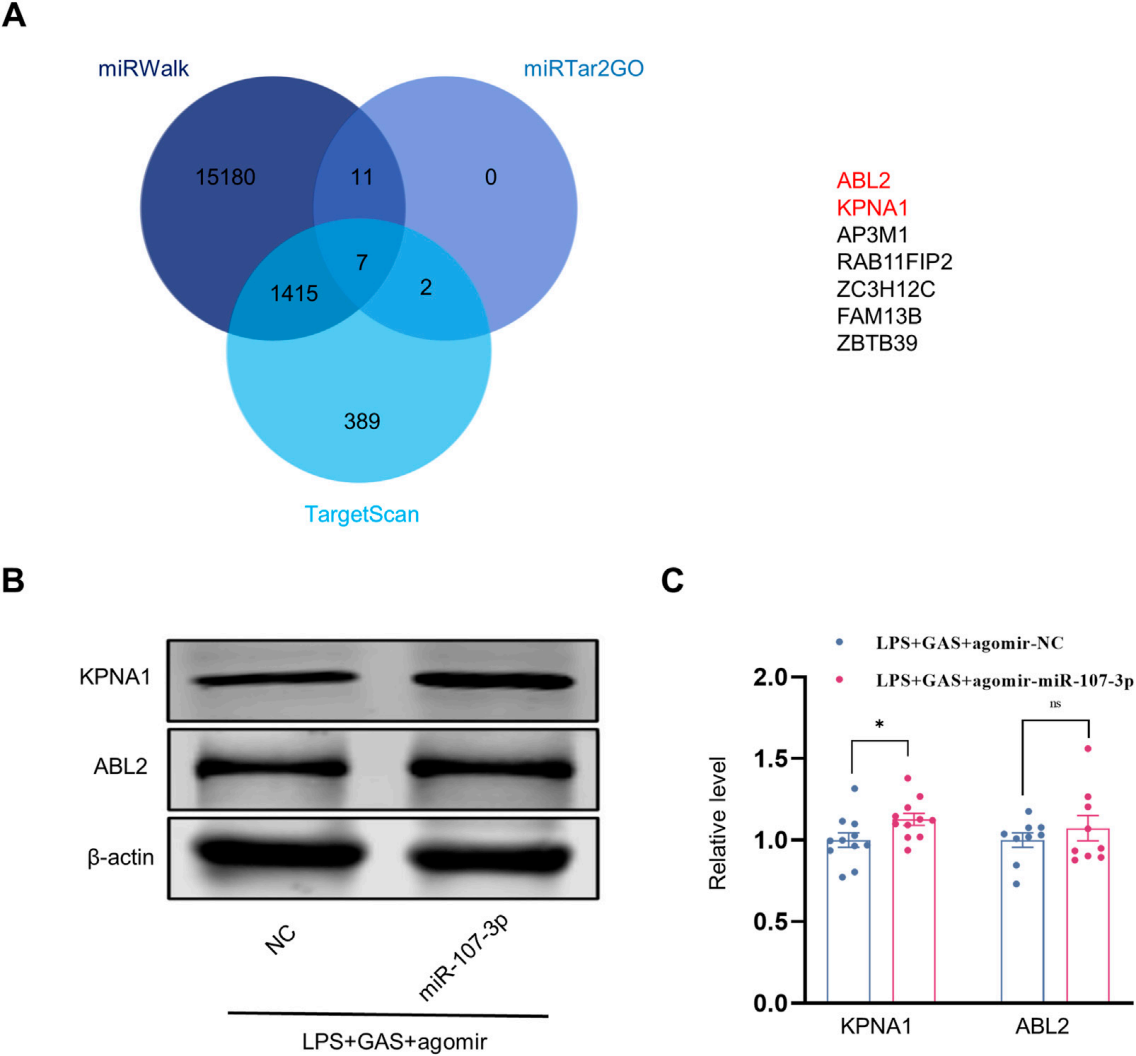
Prediction and verification of the target molecular of miR-107-3p. **(A)** Venn plots of downstream target molecules were screened by three databases (miRWalk, TargetScan, and miRTar2GO). **(B,C)** Representative protein bands and the histograms of bands intensity analysis of ABL2 and KPNA1 in the hippocampus. Values are represented as means ± SEM. *n* ≥ 9. *p < 0.05 versus LPS + GAS + agomir NC group. LPS, lipopolysaccharide; GAS, gastrodin.

To identify the target mRNA of miR-107-3p, we first used three databases (miRWalk, TargetScan, and miRTar2GO) to predict the potential mRNA of miR-107-3p. As shown in Figure 6A, seven mRNAs were found in the intersection set of three databases. Through the GTEx database query distribution in tissues and organs, we chose KPNA1 and ABL2, which are rich in the brain to conduct follow-up experiments (Supplementary Figures S3A,B). Subsequently, western blot was performed to verify the mRNA and protein expression levels. The mRNA of two predicted targets, ABL2 and KPNA1, were downregulated in the LPS + GAS + miR-107-3p agomir group compared with the LPS + GAS + agomir NC group. However, there was no statistically significant change. Meanwhile, the protein expression levels of ABL2 and KPNA1 in the miR-107-3p agomir group were also decreased compared to the control group. Among them, the changes in KPNA1 were statistically different Figures 6B,C. Therefore, the present study preliminary disclosure that KPNA1 might be involved in the neuroprotection effect of GAS on LPS-induced mice.

**FIGURE 7:**
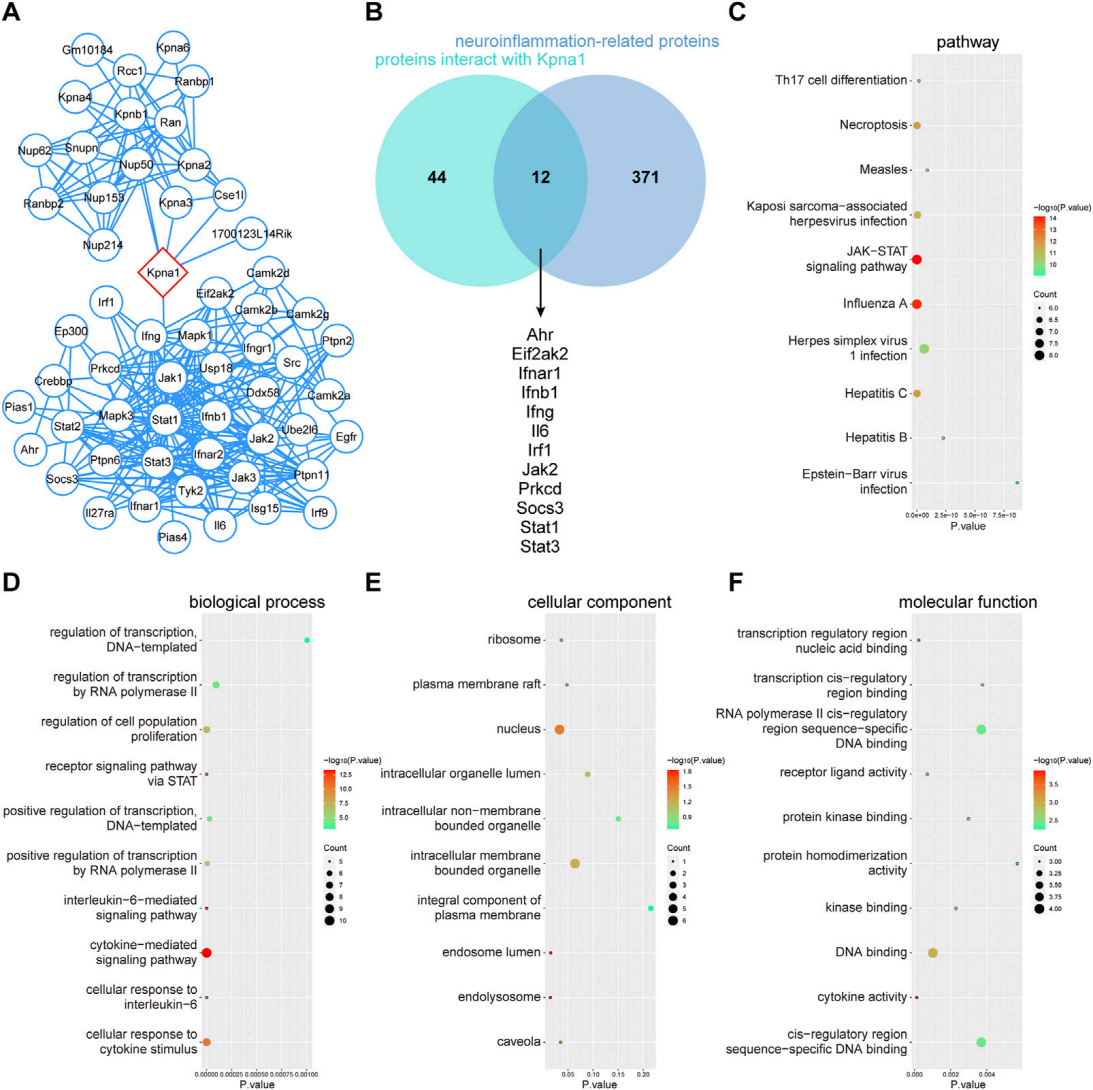
PPI network, GO terms and KEGG pathway enrichment analysis of common genes of KPNA1 and neuroinflammation. **(A)** PPI network of KPNA1 and related proteins. **(B)** Venn diagram of the intersection target of KPNA1 and neuroinflammation. **(C)** KEGG pathway enrichment analysis of 12 common genes. **(D–F)** GO function enrichment analysis of 12 common genes. Different bubble charts of biological process (BP), cellular component (CC), and molecular function (MF). The size of each circle indicates gene count. The color of circles represents different -log10 (*p-*value).

To further clarify the fuction of KPNA1, PPI network, GO and KEGG pathway enrichment analysis were performed. The enriched GO terms were divided into BP, CC, and MF. Figure 7A showed the PPI network of potential interaction proteins with core protein KPNA1. As shown in Figure 7B, a total of 56 predicted targets of KPNA1 and 383 targets for KPNA1 were intersected, and 12 potential target genes were obtained including Ahr, Eif2ak2, Ifnar1, Ifnb1, Ifng, Il6, Irf1, Jak2, Prkcd, Socs3, Stat1, and Stat3. The three most significantly enriched KEGG pathways were JAK−STAT signaling pathway, Influenza A and Necroptosis pathway (Figure 7C). Additionally, the GO analysis results indicated that DNA binding, cytokine activity and transcription regulatory region nucleic acid binding were most significantly enriched GO terms in terms of MF analysis. For the CC terms, the common genes were mainly enriched in nucleus, intracellular membrane−bounded organelle and endolysosome. Importantly, for BP analysis, the common genes were closely related to cytokine−mediated signaling pathway, cellular response to cytokine stimulus. The results obtained by enrichment analysis were illustrated by a bubble diagram (Figures 7D–F). Meanwhile, we had found that KPNA1 displayed a positive correlation with and the inflammatory factor such as KPNA1, TNF, IL-6, IL-18, and MCP1 except for IL-1β using GEPIA database (Supplementary Figures S4A–E). Therefore, KPNA1 might play an important role in neuroinflammation response.

## Discussion

Neuroinflammation is closely related to the occurrence and development of CNS disorders. A considerable number of studies suggest that peripheral inflammation can affect the brain to further induce behavioral changes such as depressive-like and anxiety-like behaviors (Cazareth et al., 2014). Here, we found that after 6 h of LPS treatment, mice were observed to exhibit obvious depressive and anxiety symptoms through behavioral evaluation. The present study used classical test methods to observe depressive-anxiety -like behavior in LPS-induced mice, including OFT, CFT, TST, FST, and EPM. In the meantime, we also noted that there were no statistical differences 24 h after LPS induction. The differences may lie in different doses and methods of LPS administration in contrast with the previous study. Thus, it could be proved that the LPS for 6 h modeling successfully established depressive-anxiety-like changes in mice. Then, we elucidated the effects of GAS in low, middle, and high doses after LPS induction. We found that middle-dose GAS could significantly ameliorate LPS-induced depressive and anxiety behaviors in mice, which was consistent with the results of previous studies (Ye et al., 2018). However, the sickness symptoms were not improved in the low-dose and high-dose GAS groups. It was probably because the low dosage of GAS didn’t work in the acute phase of brain damage and the high dosage of GAS might produce toxic side effects. These results indicated that GAS exhibited good antidepressant and antianxiety performance.

Accumulated evidence suggests that neuroinflammation, especially microglia-mediated neuroinflammation, has been shown to contribute to the pathogenesis of CNS diseases. Activation of glial cells including microglia and/or astrocytes is a prominent feature of neuroinflammation in acute brain injury, which has always been one of the focuses of biomedical research. Among them, microglia, the resident innate immune cells in the brain, become over-activated to induce neuronal damage in response to diverse stimuli. Abnormally activated glial cells promote the release of inflammatory cytokines such as TNF-α, IL-1β, and IL-6. Therefore, it was common believed that inhibiting the activation of glial cell and reducing neuroinflammation are beneficial to the improvement of depressive-like behaviors (Song et al., 2020). To explore the possible mechanism of GAS on antidepressant and antianxiety effects, we measured the expressions of pro-inflammatory cytokines as well as immunofluorescence intensity of microglia, astrocytes, and neurons in the hippocampus which is one of the key brain areas related to depression and anxiety. As expected, we found that LPS administration induced remarkably elevations of pro-inflammatory cytokines including TNF-α, IL-1β, IL-18, IL-6, and MCP-1, whereas GAS could effectively inhibit the elevations, which indicated the effect of GAS on neuroinflammation. The results are consistent with the previous studies (Li et al., 2018). Indeed, we also found that GAS simultaneously had a significant effect on the activation of microglia and astrocytes in LPS-induced mice. Unexpectedly, no effect was observed on neurons. One attractive possibility was that the mice were in an acute stage of inflammation.

As epigenetic regulators, miRNAs modulate various cellular and organ functions by regulating their target genes under different conditions (Kim et al., 2021). What’s more, miRNAs also participate in the regulation of some traditional herbs. It has been reported that miR-103, miR-21, miR-21-5p, miR-331-5p, miR-22-3p, and miR-142a regulated the protective effects of GAS in some disease models (Zhang et al., 2019; Xi et al., 2020; Shu et al., 2021; Wang and Wu, 2021). However, there are as yet no studies on miRNAs modulating GAS against neuroinflammation. To more comprehensively elucidate the underlying mechanism of GAS effects, the HTS method was adopted to evaluate the regulation of miRNA under LPS-induced brain inflammation conditions. In this study, we presented the first data to identify six abnormal miRNAs showing a major alteration in response to LPS and GAS treatment. mmu-miR-532-5p, mmu-miR-532-3p, and mmu-miR-6395 were markedly downregulated in the LPS-induced group, but upregulated after GAS treatment. mmu-miR-107-3p and mmu-miR-297-5p, mmu-miR-21a-3p were clearly upregulated in the LPS-induced group but downregulated after GAS treatment. Furthermore, miR-532-5p, miR-532-3p, and miR-21a-3p have also been reported in current literature to be directly involved in the regulation of inflammation (Zhou et al., 2019; Yan et al., 2020). However, qRT-PCR verification results showed that the various trends in the expression of miR-107-3p were basically the same as that of HTS. Regarding miR-107-3p, most previous research focused mainly on cancers such as glioma. Interestingly, miR-107-3p on brain diseases acting as positive or negative regulators have been controversial. In the study, we found that miR-107-3p increased after LPS short-term induction, indicating miR-107-3p may promote brain injury reversed by GAS. Yang et al. also argued that miR-107-3p inhibition can protect the rat cerebrum from excitatory neurotoxicity during ischemia-reperfusion injury (Yang et al., 2014; Yang et al., 2015). On the contrary, it has been reported that miR-107 in analogy with miR-107-3p can protect against knee osteoarthritis by downregulating caspase-1 to decrease pyroptosis (Qian et al., 2021). Such inconsistency or discrepancies between these studies may be explained in part by the fact that differences in tissue sources, handling time and method, techniques used for miRNA profiling et al.

To further investigate the results of HTS and further explore the relationship between miR-107-3p and the effects of GAS, we constructed a miR-107-3p agomir to determine its potential roles. It was observed that miR-107-3p overexpression could reverse the anti-depressive and anti-anxiety like behaviors and anti-inflammatory effects of GAS. The significance of this finding reveals the fact that miR-107-3p plays a crucial role in the neuroprotection of GAS. Therefore, inhibition of miR-107-3p may represent a new potential therapeutic target for inflammation-related brain disorders. Prediction of targets of miRNAs may assist in the understanding of the regulatory role of abnormal miRNAs. Some reseaches indicated that CDK5R1, GRN, or Dicer1 were identified as a direct target of miR-107 (Wang et al., 2010; Moncini et al., 2011; Li et al., 2015). Finally, we consulted three databases simultaneously to predict targets mRNA of miR-107-3p. For all three algorithms, ABL2 (tyrosine-protein kinase ABL2) and KPNA1 (Importin subunit α5), which are enriched in the brain, were the shared predicted targets. At present, no studies have documented the direct effects of miR-107-3p on the two targets. However, bioinformatic predictions for targets must be subjected to further verification *via in vivo* experiments. Meanwhile, we also examined the mRNA and protein levels of target molecules by qRT-PCR and western blot. We found that overexpression of miR-107-3p could inhibit the mRNA expression levels of KPNA1 and ABL2, whereas it could promote the protein expression of KPNA1 and ABL2. Among them, there was a significant difference in KPNA1 protein expression. KPNA1, as an adapter protein for nuclear receptor KPNB1, mainly participates in protein import into nucleus and regulation of apoptotic process (Choo et al., 2016). It is worth confused that KPNA1 mRNA expression decreased, but protein expression level increased. It is speculated that the possible reason is that mir-107-3p is not bound to the classical 3′-UTR region of KPNA1 mRNA, or the stability of KPNA1 protein increases which has less formation and less degradation. However, the potential function of KPNA1 on neuroinflammation is well unknown. Thus, we preliminary carried out PPI, GO cluster analysis and KEGG pathway analysis to predict its function. Based on the bioinformatics analysis we found that KPNA1 and related proteins on neuroinflammation could regulate JAK−STAT signaling pathway, Influenza A and Necroptosis pathway et al., which will provide a theoretical basis for comprehensive and futher research.

The main weakness of our study is the failure to address the interaction between miR-107-3p and its targets. More experiments should be undertaken to further explore the more close links such as RNA pull-down and luciferase reporter assays. Subsequently, the functional impact of KPNA1 in the regulation of GAS on neuroinflammation *in vivo* further research should be further explored by more accurate future researches. Finally, the necessary correlation between miR-107-3p and GAS still needs to be confirmed with miR-107-3p knockout mice.

**FIGURE 8:**
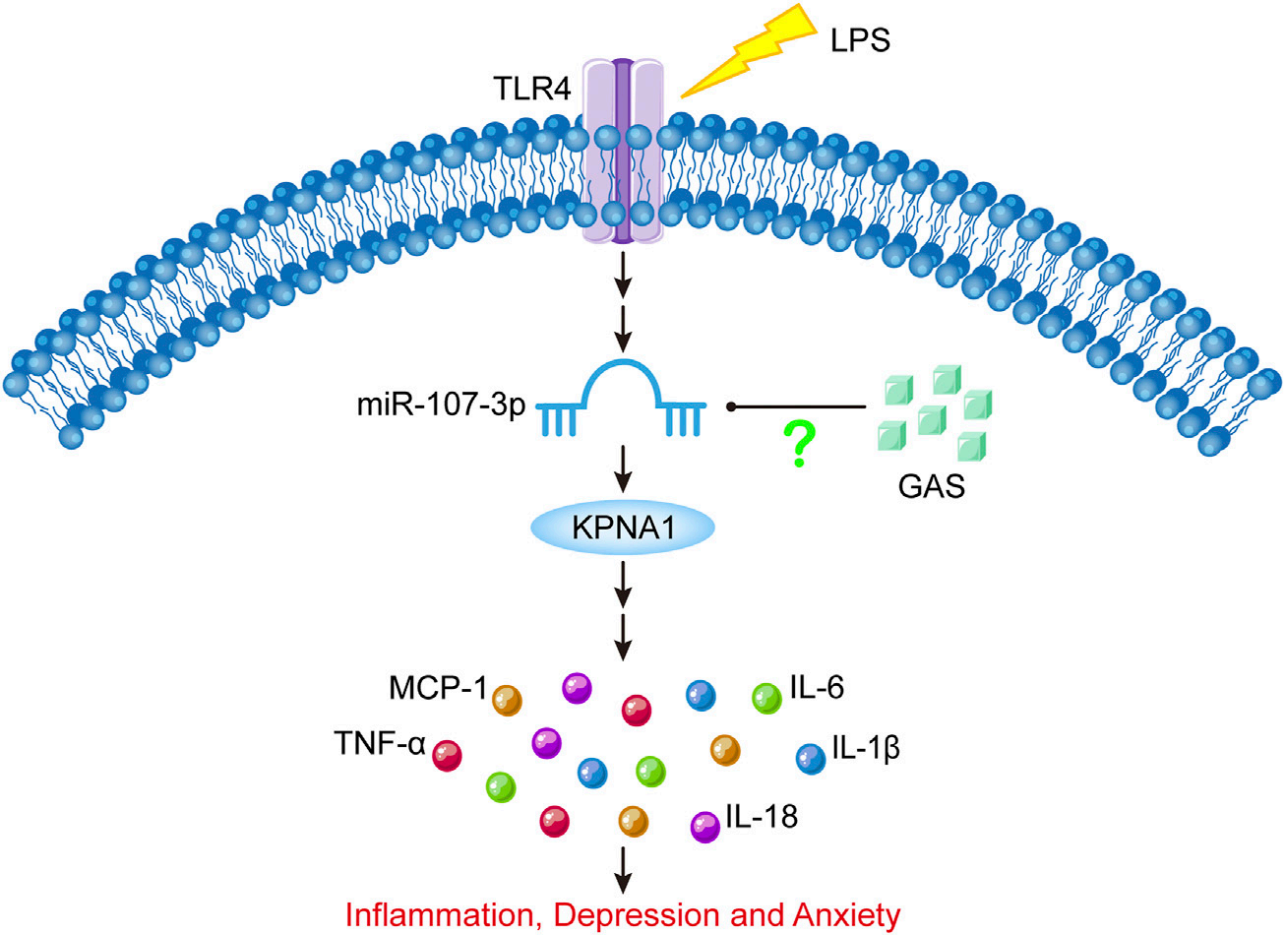
Schematic diagram of the present experimental results. LPS stimulation can increase miR-107-3p, thus promoting the expression of KPNA1, and ultimately promoting the production of inflammatory cytokines, which leads to inflammatory response and depressive-like behavior. GAS can downregulate the expression of KPNA1 by inhibiting miR-107-3p, reducing the production of inflammatory factors, and alleviating inflammatory response and depressive-like behavior. LPS: lipopolysaccharide; GAS, gastrodin; KPNA1, Karyopherin Subunit Alpha1; IL-1β, Interleukin-1; IL-6, Interleukin-6; IL-18, Interleukin-18; Tumor necrosis factor-α, TNF-α; MCP-1, Monocyte Chemoattractant Protein-1; TLR4, toll-like receptor 4.

All in all, our results suggested that GAS might ameliorate neuroinflammation and depressive-anxiety-like behaviors in LPS-induced mice by downregulating miR-107-3p and the downstream target KPNA1, which are shown in the schematic diagram Figure 8. These findings provided an experimental basis for the clinical application of GAS in the treatment of neuroinflammatory-related CNS disorders.

## Data Availability

The datasets presented in this study can be found in online repositories. The names of the repository/repositories and accession number(s) can be found in the article/Supplementary Material.

## Supplementary material

SUPPLEMENTARY FIGURE S1 Effects of GAS or miR-107-3p on neurons in the hippocampal region of mice. **(A)** images of IF staining for NeuN-positive neuron (red) in the CA1, CA2, CA3, and DG regions of hippocampus after LPS and GAS treatments. DAPI-blue. Scale bar = 60 μm. **(B)** HE staining images for pyramidal neurons in the hippocampal region after LPS and GAS treatments. Scale bar = 100 μm. **(C)** images of IF staining for NeuN-positive neuron (red) in the hippocampus of mice after miR-107-3p agomir injection. DAPI-blue. Scale bar = 60 μm. LPS, lipopolysaccharide; GAS, gastrodin.

SUPPLEMENTARY FIGURE S2 Diffusion conditions of miR-107-3p in the lateral ventricle and hippocampus of mice. **(A)** bromophenol blue stereotaxic injection in the lateral ventricle and hippocampus of mice. **(B)** Sequence diagram of agomir miR-107-3p and NC. **(C)** Schematic diagram tereotaxic injection and fluorescence expression map of miR-107-3p in the hippocampus and lateral ventricle of mice after 6, 12, and 24 h injection. scale bar = 120 μm. LPS, lipopolysaccharide; GAS, gastrodin.

SUPPLEMENTARY FIGURE S3 Expression maps of ABL2 and KPNA1 in tissues and organs.

SUPPLEMENTARY FIGURE S4 The potential correlation analysis between KPNA1 and the inflammatory factor (TNF, IL-6, IL-1β, IL-18 or MCP1) using GEPIA database. **(A-E)** The potential correlation analysis diagram between KPNA1 and TNF, IL-6, IL-1β, IL-18 or MCP1, respectivly. Pearson correlation analysis of *R* and *P*-values were used.

SUPPLEMENTARY TABLE S1 Primer sequences used in the quantitative RT-PCR analysis.

SUPPLEMENTARY TABLE S2 miRNA expression data generated by High-throughput Sequencing (HTS) analysis.
