# Supplementary figures and images for: Gastrodin ameliorates the lipopolysaccharide-induced neuroinflammation in mice by downregulating miR-107-3p
Source: Front Pharmacol. 2022 Dec 8;13:1044375. doi: 10.3389/fphar.2022.1044375 (PMC9773390; doi:10.3389/fphar.2022.1044375)

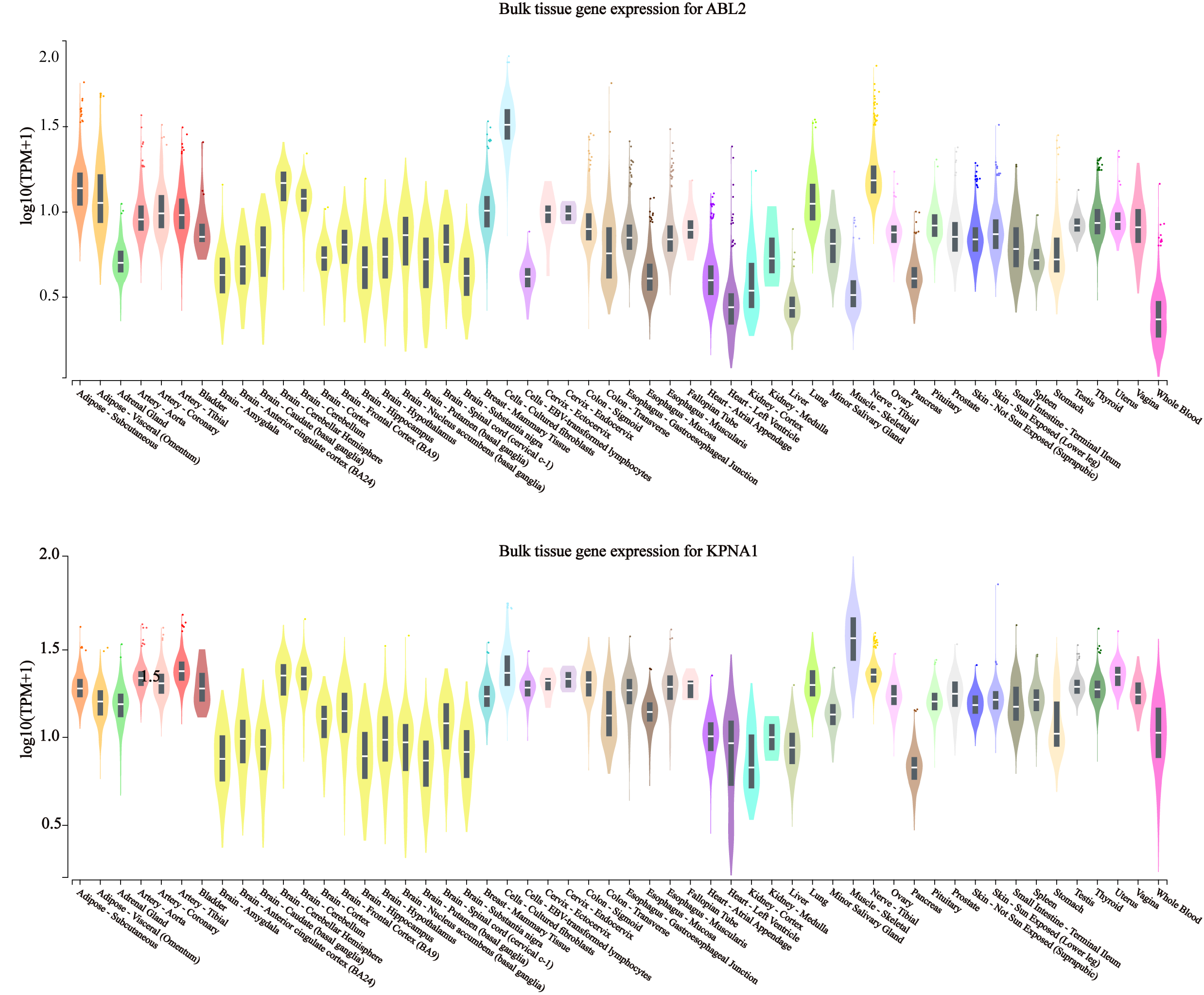

Supplement: Supplementary file 1 [file Image3.TIF]

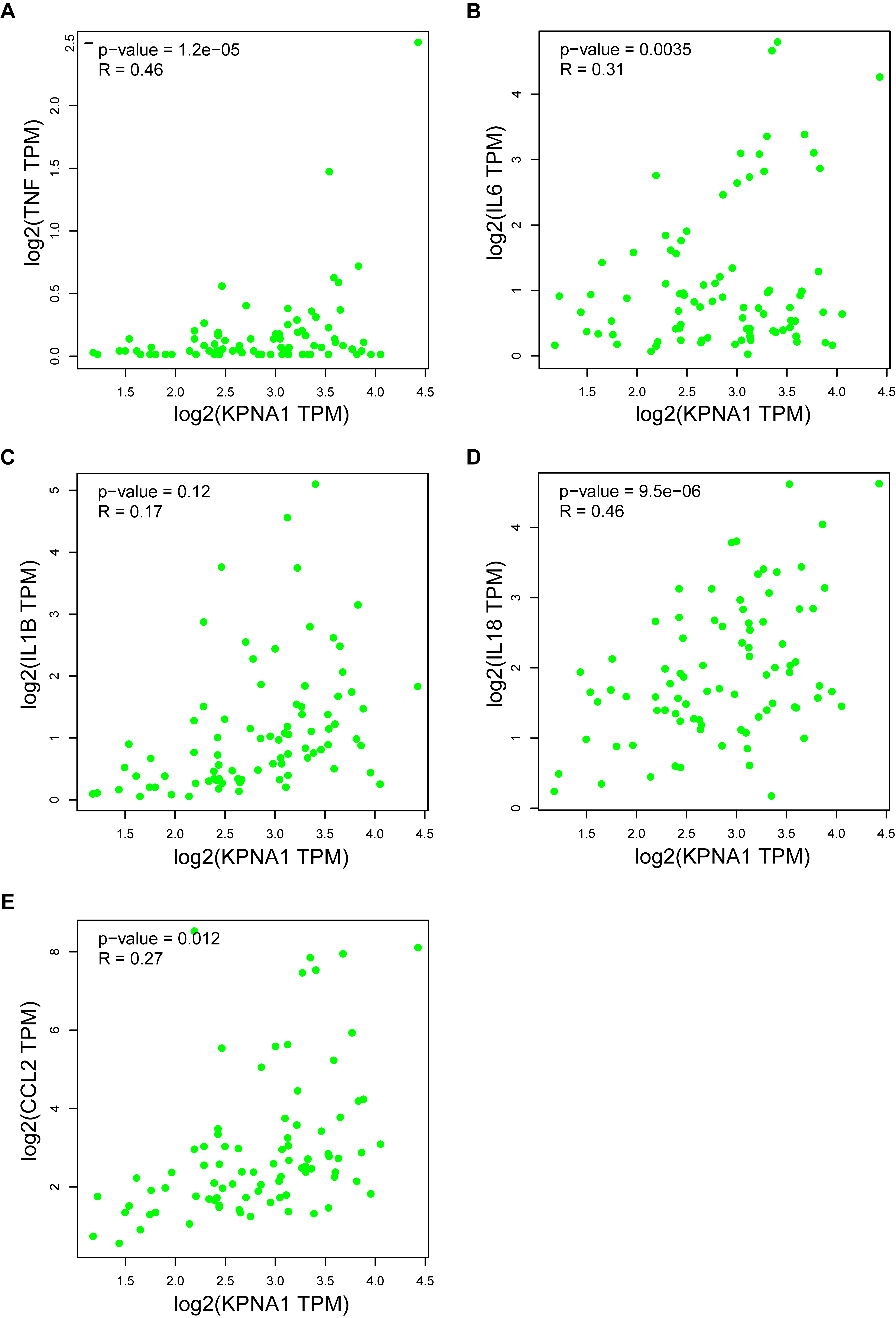

Supplement: Supplementary file 2 [file Image4.TIF]

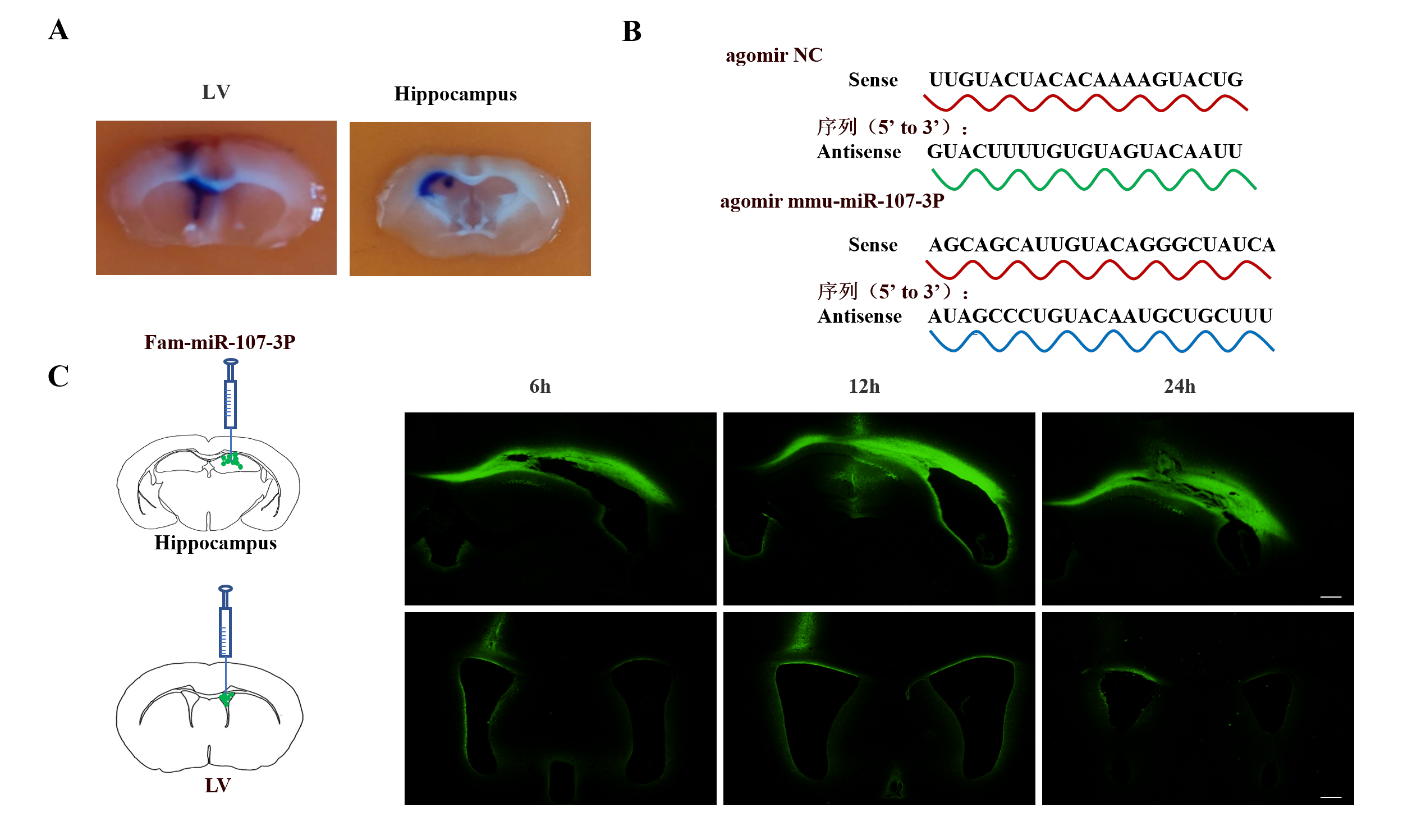

Supplement: Supplementary file 3 [file Image2.TIF]

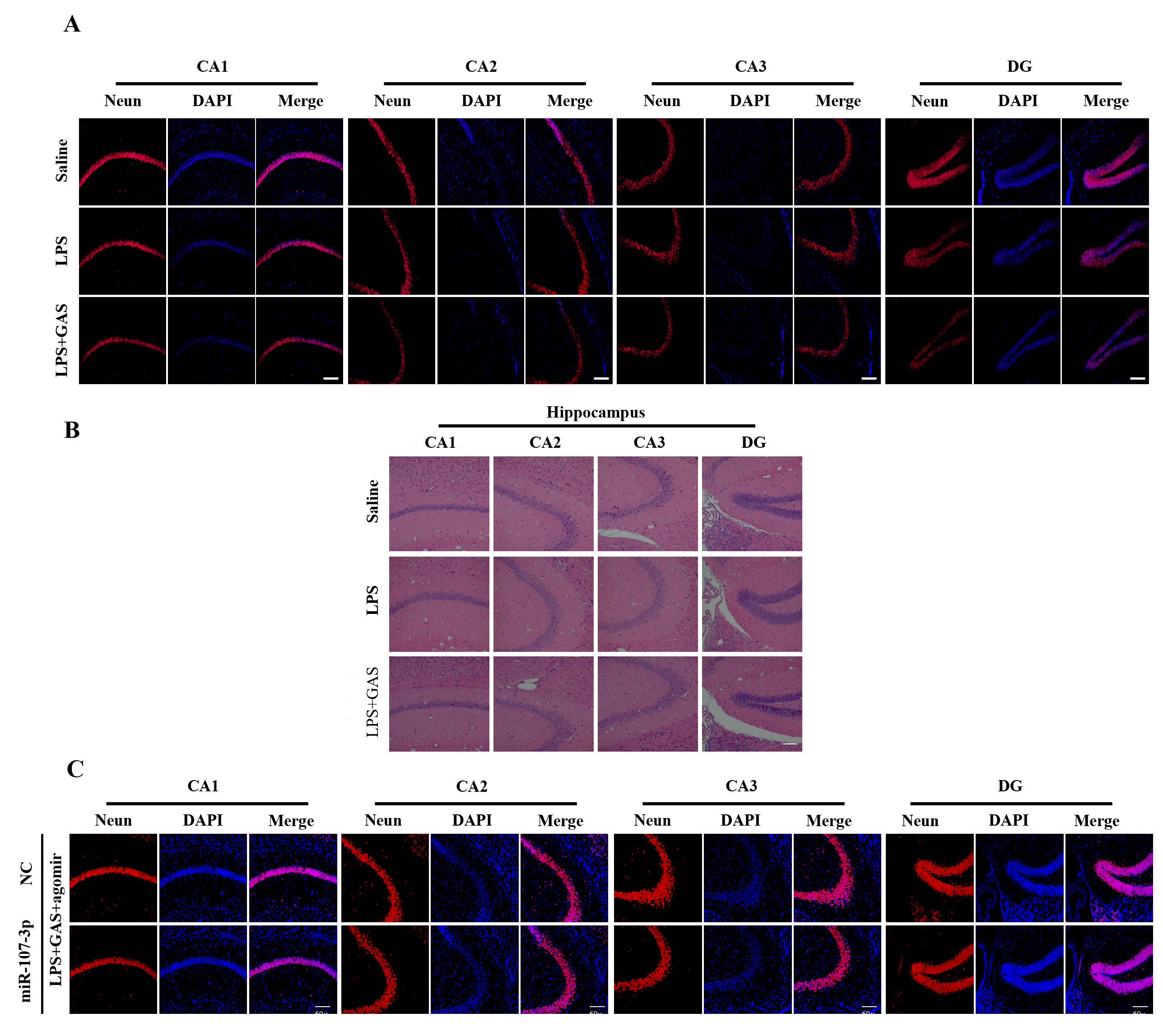

Supplement: Supplementary file 4 [file Image1.TIF]
